# Relating Neuronal Firing Patterns to Functional Differentiation of Cerebral Cortex

**DOI:** 10.1371/journal.pcbi.1000433

**Published:** 2009-07-10

**Authors:** Shigeru Shinomoto, Hideaki Kim, Takeaki Shimokawa, Nanae Matsuno, Shintaro Funahashi, Keisetsu Shima, Ichiro Fujita, Hiroshi Tamura, Taijiro Doi, Kenji Kawano, Naoko Inaba, Kikuro Fukushima, Sergei Kurkin, Kiyoshi Kurata, Masato Taira, Ken-Ichiro Tsutsui, Hidehiko Komatsu, Tadashi Ogawa, Kowa Koida, Jun Tanji, Keisuke Toyama

**Affiliations:** 1 Graduate School of Science, Kyoto University, Sakyo-ku, Kyoto, Japan; 2 Kokoro Research Center, Kyoto University, Sakyo-ku, Kyoto, Japan; 3 Department of Physiology, Tohoku University School of Medicine, Aoba-ku, Sendai, Japan; 4 Laboratory for Cognitive Neuroscience, Graduate School of Frontier Biosciences, Osaka University, Toyonaka, Osaka, Japan; 5 Department of Integrative Brain Science, Graduate School of Medicine, Kyoto University, Sakyo-ku, Kyoto, Japan; 6 Department of Physiology, Hokkaido University School of Medicine, Sapporo, Japan; 7 Department of Physiology, Hirosaki University School of Medicine, Hirosaki, Japan; 8 Division of Applied System Neuroscience, Advanced Medical Research Center, Nihon University Graduate School of Medical Science, Tokyo, Japan; 9 Division of Systems Neuroscience, Tohoku University Graduate School of Life Sciences, Aoba-ku, Sendai, Japan; 10 Division of Sensory and Cognitive Information, National Institute for Physiological Sciences, Myodaiji, Okazaki, Aichi, Japan; 11 Tamagawa University Brain Science Institute, Machida, Tokyo, Japan; 12 ATR Computational Neuroscience Laboratories, Seika-cho, Soraku-gun, Kyoto, Japan; John Radcliffe Hospital, United Kingdom

## Abstract

It has been empirically established that the cerebral cortical areas defined by Brodmann one hundred years ago solely on the basis of cellular organization are closely correlated to their function, such as sensation, association, and motion. Cytoarchitectonically distinct cortical areas have different densities and types of neurons. Thus, signaling patterns may also vary among cytoarchitectonically unique cortical areas. To examine how neuronal signaling patterns are related to innate cortical functions, we detected intrinsic features of cortical firing by devising a metric that efficiently isolates non-Poisson irregular characteristics, independent of spike rate fluctuations that are caused extrinsically by ever-changing behavioral conditions. Using the new metric, we analyzed spike trains from over 1,000 neurons in 15 cortical areas sampled by eight independent neurophysiological laboratories. Analysis of firing-pattern dissimilarities across cortical areas revealed a gradient of firing regularity that corresponded closely to the functional category of the cortical area; neuronal spiking patterns are regular in motor areas, random in the visual areas, and bursty in the prefrontal area. Thus, signaling patterns may play an important role in function-specific cerebral cortical computation.

## Author Summary

Neurons, or nerve cells in the brain, communicate with each other using stereotyped electric pulses, called spikes. It is believed that neurons convey information mainly through the frequency of the transmitted spikes, called the firing rate. In addition, neurons may communicate some information through the finer temporal patterns of the spikes. Neuronal firing patterns may depend on cellular organization, which varies among the regions of the brain, according to the roles they play, such as sensation, association, and motion. In order to examine the relationship among signals, structure, and function, we devised a metric to detect firing irregularity intrinsic and specific to individual neurons and analyzed spike sequences from over 1,000 neurons in 15 different cortical areas. Here we report two results of this study. First, we found that neurons exhibit stable firing patterns that can be characterized as “regular”, “random”, and “bursty”. Second, we observed a strong correlation between the type of signaling pattern exhibited by neurons in a given area and the function of that area. This suggests that, in addition to reflecting the cellular organization of the brain, neuronal signaling patterns may also play a role in specific types of neuronal computations.

## Introduction

Neurons transmit stereotypical electrical pulses called spikes. The *in vivo* spike firing of cortical neurons is often regarded as a series of simple random events that conveys no information other than the frequency, or rate, of occurrences. However, it is possible that neuronal firing patterns differ between brain regions, because biological, as well as mechanical, signals generally reveal internal conditions of the signal generator. It has been known for a century that the cellular organization of the brain is not homogeneous [1], and areas categorized on cytoarchitectonic bases govern different functions [2]–[4]. Therefore, temporal signaling patterns of neurons may reflect the cellular organization and also effectively control specific computations [5]–[12]. In order to examine the relationship among signals, structure, and function, we analyzed spike trains sampled from various brain regions.

A number of studies have been devoted to analysis of interspike interval (ISI) distributions of firing patterns, and sophisticated analyses have shown that *in vivo* neuronal firing is not exactly a random Poisson phenomenon [13]–[23]. However, analysis of raw ISIs is vulnerable to fluctuations in the firing rate that scatter the ISI values; even temporally regular spike trains tend to be evaluated closer to the faceless Poisson random sequence. This perturbation, which is extrinsic in origin, can be removed by rescaling ISIs with the instantaneous firing rate [24]–[31].

Previously, we devised a metric of local variation, *Lv*, which may straightforwardly isolate instantaneous firing regularity or irregularity. We found that for individual neurons, the degree of firing irregularities is fairly invariant with time and rate fluctuations [32],[33]. In contrast, it was reported that another metric, *IR*, which measures the instantaneous irregularity similar to *Lv*, varies in time and with behavioral context [34]. Thus, current analysis methods are still inadequate for extracting intrinsic firing characteristics in isolation from extrinsic perturbations.

Here, we have derived a new metric, *LvR*, by enhancing the invariance to firing rate fluctuations, such that signaling characteristic that are specific to individual neurons can be detected with greater sensitivity. We analyzed differences in intrinsic firing characteristics among the cortical areas and found a systematic gradient of firing regularity that closely corresponded with the functional category of the cortical area; neuronal firing is relatively regular in primary and higher-order motor areas, random in visual areas, and bursty in the prefrontal area. Thus, intrinsic dynamics are present in cortical areas that may be relevant to function-specific cortical computations.

## Materials and Methods

### Spike Data Analysis

Neuronal data for 15 cortical areas were collected from awake, behaving monkeys in eight laboratories. Four of the 15 areas were studied in two laboratories, thus 19 data sets were generated in total. Single electrodes or tetrodes were used to record neuronal spikes during various task trials and inter-trial intervals. All procedures for animal care and experimentation were in accordance with the guidelines of the National Institutes of Health and approved by the animal experiment committee at the respective institution where the experiments were performed.

The initial 2,000 ISIs of the recorded spike train for each neuron were analyzed, which contained task trial periods and inter-trial intervals, between which the firing rate differs greatly. Spike trains that contained fewer than 2,000 ISIs, or those with mean firing rates less than 5 spikes/s, were ignored; 1,307 neurons were accepted. An irregularity metric was computed for the entire 2,000 ISIs to yield a representative value for each neuron. They are divided into 20 sequences of 100 ISIs for analyzing fractional sequences; the variation of a metric for an individual neuron was estimated by comparing metric values computed for 20 fractional sequences.

### Firing Metrics

Six firing metrics were used to analyze the spike data.

The conventional coefficient of variation *Cv* [35],[36] is defined as the ratio of the standard deviation of the ISIs 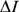 to the mean 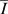,
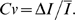
(1)The local variation *Lv* [32],[33] is defined as
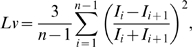
(2)where 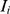 and 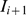 are the *i*-th and *i*+1st ISIs, and *n* is the number of ISIs. Both *Cv* and *Lv* adopt a value of 0 for a sequence of perfectly regular intervals and are expected to take value of 1 for a Poisson random series of events with ISIs that are independently exponentially distributed. Whereas *Cv* represents the global variability of an entire ISI sequence and is sensitive to firing rate fluctuations, *Lv* detects the instantaneous variability of ISIs: The term 
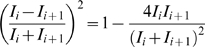
 represents the cross-correlation between consecutive intervals 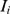 and 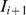, each rescaled with the instantaneous spike rate 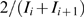. The metric is superior to standard correlation analysis because (i) the irregularity is measured separately from the firing rate; (ii) nonstationarity is eliminated by rescaling intervals with the momentary rate; and (iii) the non-Poisson feature is evaluated in the deviation from *Lv* = 1. Three more metrics that have been proposed for estimation of instantaneous ISI variability, *SI*, the geometric average of the rescaled cross-correlation of ISIs [37],[38], *Cv2*, the coefficient of variation for a sequence of two ISIs [39], and *IR*, the difference of the log ISIs [34] were also used.

**Figure 1:**
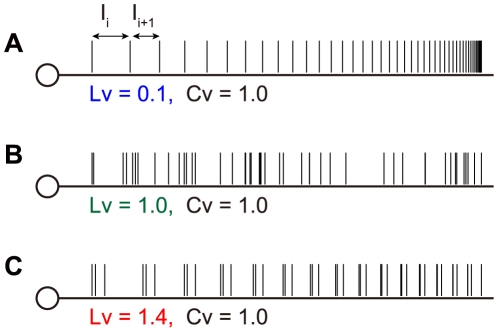
Spike sequences that have identical sets of inter-spike intervals. Intervals are aligned (A) in a regular order, (B) randomly, and (C) alternating between short and long.

Figure 1 displays three types of spike sequences comprising identical sets of exponentially distributed ISIs. In terms of the ISI distributions, all of these are regarded as Poisson processes, accordingly *Cv* values are all identical at 1. However, these sequences clearly differ in how their ISIs are arranged; *Lv* may be able to detect these differences.

In comparison with *Cv*, local metrics, such as *Lv*, *SI*, *Cv2*, and *IR*, detect firing irregularities fairly invariantly with firing rate fluctuations. However, these metrics are still somewhat dependent on firing rate fluctuations. Assuming that rate dependence is caused by the refractory period that follows a spike, we can compensate for refractoriness by subtracting the refractoriness constant, *R*, from the ISIs. As a result, the denominator of Equation 2, 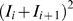 changes to 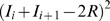. In order to avoid the singularity that may occur when 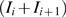 is equal to or less than 2*R*, we performed a series expansion to the first order in *R*. The revised local variation *LvR* is thus defined as
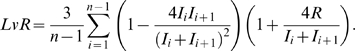
(3)


### Performance Evaluation of Firing Metrics

We evaluated how the metric performed in discrimination of individual neuronal firing patterns by the *F*-test statistic, which compares the variance of the metric means across 1,307 neurons to the mean of the metric variances across 20 fractional sequences of individual neurons. *LvR* contains the refractoriness constant, *R*, which is the parameter to be optimized to maximize characterization of firing dynamics of the individual neurons in terms of the *F*-value. For a given set of metric values 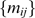, each of which is computed for *j*-th fragmental ISI sequence (*j* = 1, 2, …, *n* ( = 20)) recorded from *i*-th neuron (*i* = 1, 2, …, *N* ( = 1,307)), the *F*-value is given by
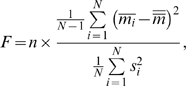
(4)where 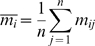 and 
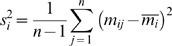
 represent the mean and variance, respectively, of the metric values of *i*-th neuron averaged over n = 20 fragments, and 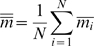 represents the average of 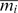 over N = 1,307 neurons.

We estimated the firing rate dependence of the metric as a covariate with firing rate fluctuations, or the slopes of the regression lines for the metric estimates.
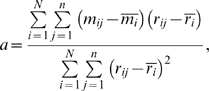
(5)where 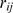 is the mean rate of *j*-th fragmental ISI sequence recorded from *i*-th neuron.

We also measured the ability of the metric to characterize the individual neuronal firing dynamics in isolation from the firing rate dependence (*F*-value of an analysis of covariance, ANCOVA, see Reference [40] for details).

### The Hellinger Distance

We found that *LvR* distributions broadly diverge across neuronal data sets. The (dis)similarity of the *LvR* distributions between two neuronal data sets is estimated as the Hellinger distance [41],
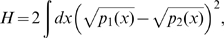
(6)where 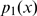 and 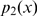 represent the normalized distributions of *LvR* values for two data sets. We feature the firing irregularity of the individual neuronal data sets as a set of Hellinger distances for all combinations of data sets (K(K−1)/2, K = 19). Kruskal's nonmetric multidimensional scaling (MDS) analysis [42] was used to contract the multidimensional features down to a two-dimensional map of firing irregularities. Here, *LvR* distributions are shown as histograms with a common bin size 0.25. The results are robustly against the choice of bin size.

## Results

Nineteen neuronal spike data sets from 15 cortical areas were collected from neuroscience experiments in awake, behaving monkeys conducted in eight laboratories. The cortical areas included the primary motor (M1), dorsal and ventral premotor (PMd and PMv), supplementary motor (SMA, two data sets from two different laboratories), presupplementary motor (preSMA), rostral cingulated motor (CMAr), supplementary eye field (SEF), frontal eye field (FEF), caudal intraparietal (CIP), striate (V1), extrastriate-dorsal stream (MT and MST, two data sets), extrastriate-ventral stream (V4 and TE, two data sets), and prefrontal areas (PF, two data sets) [32], [43]–[52] (Table 1). The neuronal firing characteristics of 1,307 neurons from the 19 data sets were analyzed using the six firing metrics, *LvR*, *Lv*, *IR*, *Cv2*, *SI*, and *Cv*. (cf. Materials and Methods).

**Table 1:** List of the cortical areas, experimental attributes and references for neuronal spike data (in order of ascending mean *LvR*).

No.	Cortical area	Functional category	No. of neurons	*LvR*		sp/s		Reference
				Mean	SD	mean	SD	
1	M1	Primary motor	26	0.51	0.34	23.2	13.9	[43]
2	SMA	Higher-order motor	83	0.57	0.34	20.4	11.0	New
3	PMd	Higher-order motor	188	0.69	0.43	20.5	12.5	[43]
4	SEF	Higher-order motor	100	0.69	0.32	15.2	6.2	[44]
5	PMv	Higher-order motor	30	0.70	0.36	26.6	18.4	[43]
6	CMAr	Higher-order motor	27	0.79	0.30	16.1	8.6	[32]
7	FEF	Higher-order motor	45	0.83	0.25	19.7	7.8	[45]
8	PreSMA	Higher-order motor	119	0.86	0.35	14.9	9.0	[32]
9	SMA	Higher-order motor	27	0.88	0.35	20.0	12.7	[32]
10	TE	Visual	102	0.88	0.30	13.8	11.6	[46]
11	MST	Visual	76	0.96	0.40	17.9	8.6	[47]
12	TE	Visual	62	0.97	0.29	13.0	11.6	New
13	V1	Visual	35	1.01	0.30	29.3	12.2	[48]
14	MST	Visual	94	1.14	0.37	17.7	9.0	[49]
15	V4	Visual	29	1.15	0.32	16.3	10.6	[50]
16	PF	Prefrontal	21	1.19	0.22	28.8	12.9	[32]
17	PF	Prefrontal	36	1.26	0.24	14.0	6.1	[51]
18	CIP	Visual	150	1.28	0.44	16.7	7.7	[52]
19	MT	Visual	57	1.39	0.33	27.7	15.4	[47]

### Discriminating Firing Patterns across Individual Neurons

**Figure 2:**
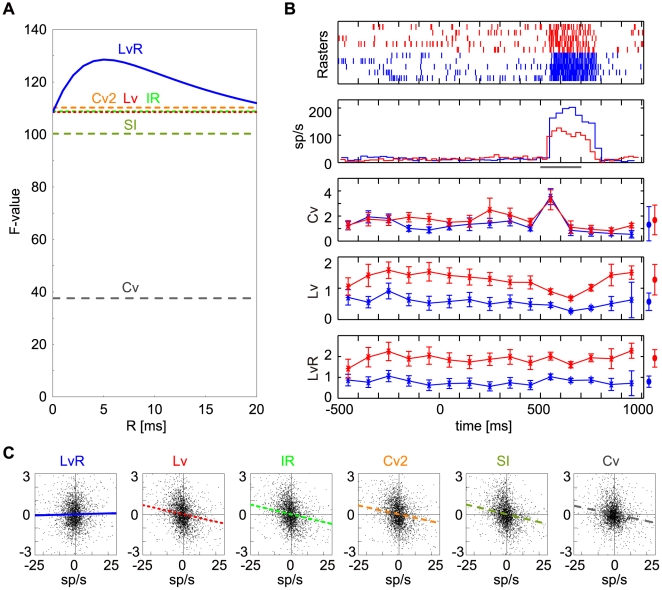
Performance of *LvR* and other firing irregularity metrics. (A) Dependence of *LvR* on the refractoriness constant, *R*. Ordinate, performance of *LvR* estimated as the *F*-value of ANOVA for the entire 1,307 neurons. (B) Peristimulus spike rasters and histograms for two MT neurons during a stimulus with textured image motion from 500–700 ms (thick horizontal bar) [47]. The spike rasters were aligned to the onset of a fixation target (the origin of the abscissa) upon which the monkey was required to fixate. The perievent metrics *Cv*, *Lv* and *LvR* were determined for spike rasters sampled in time windows of ±50 ms around the time of each bin. Error bars indicate the confidence level (p<0.05, t-distribution). (C) Scatter plots of the six metrics plotted against fluctuation in the firing rate across 20 ISI segments from the 1,307 neurons. The ordinate and abscissa represent the deviations of the metric and the firing rate from the means, normalized to SD, respectively. Colored lines represent average slopes.

Although *LvR* is primarily designed to strengthen the firing rate invariance for detection of instantaneous firing irregularities (cf. Materials and Methods, Equation 3), it may also be superior for discrimination of individual neuronal firing patterns. We evaluated metric performance using the *F*-test statistic, which compares the variance of the metric means across neurons to the mean of the metric variances across fractional sequences of individuals (cf. Materials and Methods, Equation 4); metrics with higher *F*-values are better able to distinguish neurons with different spiking patterns. Figure 2A shows the performance of the six metrics. The *F*-value is low (*F* = 38) for *Cv* and is greater for *Lv*, *IR*, *Cv2*, and *SI* (*F* = 109, 109, 110, and 100, respectively). *LvR* is a function of *R* and is greatest (*F* = 129) for *R* = 5 ms. Thus, we used *R* = 5 ms to analyze all of the neuronal data.

In practice, the optimized *LvR* exhibits the strongest invariance with the firing rate, as shown for two representative MT neurons (Figure 2B, red and blue traces). Both neurons responded strongly to texture motion (black bar under the spike rate plot), the firing rate increased roughly 10-fold (108±11 and 189±14 spikes/s) over baseline (13.0±5.4 and 12.6±4.8, respectively). Correspondingly, *Cv* increases roughly two-fold and is then reduced to half the baseline. *Lv* is reduced to roughly two-thirds of the baseline. *IR*, *Cv2*, and *SI* also exhibit a dependence on the firing rate comparable to *Lv* (data not shown). By contrast, *LvR* maintains values unique to each of the two neurons throughout the entire sampling period and is virtually unaffected by large changes in the firing rate.

Regression analysis to estimate the firing rate dependence as a covariate of the metric estimates with firing rate fluctuations across 20 fractional ISI sequences for the 1,307 neurons (cf. Materials and Methods, Equation 5) also confirms that *LvR* is one order of magnitude better in invariance (slope and 95% confidence interval, 0.0033±0.0012 [sec], cf. also solid blue line in Figure 2C) than *Lv*, *IR*, *Cv2*, *SI*, and *Cv* (−0.0273±0.0012, −0.0287±0.0012, −0.0261±0.0012, −0.0289±0.0012, and −0.0254±0.0012, respectively, [sec], cf. also dashed lines in Figure 2C). The analysis of covariance (ANCOVA) indicates that *LvR* performs better (*F* = 129) than *Lv*, *IR*, *Cv2*, *SI*, and *Cv* (*F* = 115, 115, 116, 106, and 40, respectively) for discrimination of individual neuronal firing dynamics even allowing for the firing rate dependence. Therefore, introduction and optimization of the refractory term in *LvR* improves characterization of neuronal firing dynamics more than compensating for the firing rate dependence.

Overall, *LvR* with *R* = 5 ms far outperforms the other five metrics for characterization of neuronal firing dynamics because it assigns unique values to individual neurons that are preserved across extrinsic perturbations such as firing rate fluctuations and sensory stimulation.

### Discriminating Firing Patterns across Cortical Areas

**Figure 3:**
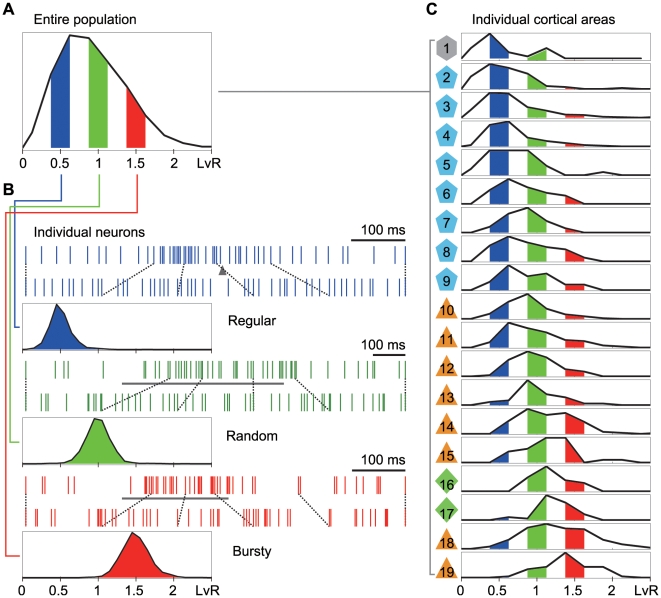
*LvR* distributions for the entire population and subpopulations of neurons. (A) The distribution of *LvR* determined across 2,000 ISI sequences for all 1,307 cortical neurons. (B) Top: Original and rescaled specimen spike sequences of representative neurons with an *LvR* of 0.5, 1.0, and 1.5 taken from data sets #2 (SMA), #10 (TE) and #19 (MT). Triangle and horizontal bars for the three original spike sequences indicate the onset of wrist movement and periods of visual stimulation, respectively. Dashed lines indicate the correspondence between the original and the rescaled fractions (10 ISIs) for which the time-scale is normalized to the average firing rate of that fraction. Bottom: Metric distributions for fractional sequences derived from neurons whose representative (mean) *LvR* values are within the range of ±0.05 around 0.5, 1.0, and 1.5 (blue, green and red bars in *A*, n = 92, 91 and 60). (C) *LvR* distributions for the 19 neuronal data sets (Table 1), shown in order of ascending mean *LvR*. The primary motor, higher-order motor, visual, and prefrontal areas are indicated as hexagons, pentagons, triangles, and squares, respectively.

Figure 3A shows the distribution of *LvR*s for the ISIs of the entire neuronal ensemble for the 19 data sets sampled from the 15 cortical areas. The distribution is rather broad, peaking around 0.7, and is slightly skewed toward lower values (mean±SD, 0.92±0.43). Figure 3B displays the distribution of *LvR* values for the 20 fractional sequences of 100 ISIs derived from individual neurons with a mean *LvR* (over 20 fractional sequences) exhibiting 0.5, 1.0, and 1.5 (±0.05). The fractional sequence of 100 ISIs derived from individual neurons are narrowly distributed around the mean. Their SDs are 0.13, 0.16, and 0.18, which are considerably smaller than that of the entire population (SD: 0.46). The small variation of *LvR* for the fractional sequences for each neuron indicates that *LvR* successfully captures the firing characteristics that are specific to an individual neuron.

Sample firing patterns of the three different *LvR* values corresponding to the so-called regular, random, and bursty firing patterns are also shown in Figure 3B. These patterns are maintained across time, with invariance for large changes in the firing rate caused by stimulus or behavioral modulation, i.e., regular remains regular despite large firing rate modulations (cf. the original and time-rescaled spike rasters for the mean firing rate shown at the top and bottom, respectively in Figure 3). Thus, the broad distribution of *LvR* across the entire neuronal population is made up of constituent neurons with a relatively narrow distribution that peaks across a broad range of *LvR*, and the variety of firing patterns that are observed across the entire neuronal population is constructed of a broad spectrum of constituent neurons whose firing pattern is rather sharply constrained either to regular, random, or bursty.

Accordingly, the diverse distribution of *LvR* for the entire neuronal population also consists of a spectrum of *LvR* distributions for each of the 19 neuronal data sets. These are shown in Figure 3C in order of ascending average values. It is notable that the distributions for the individual data sets are moderately broad, narrower than that of the entire neuronal population (cf. Figure 3A and 3C) but broader than those of individual neurons (cf. Figure 3B and 3C).

**Figure 4:**
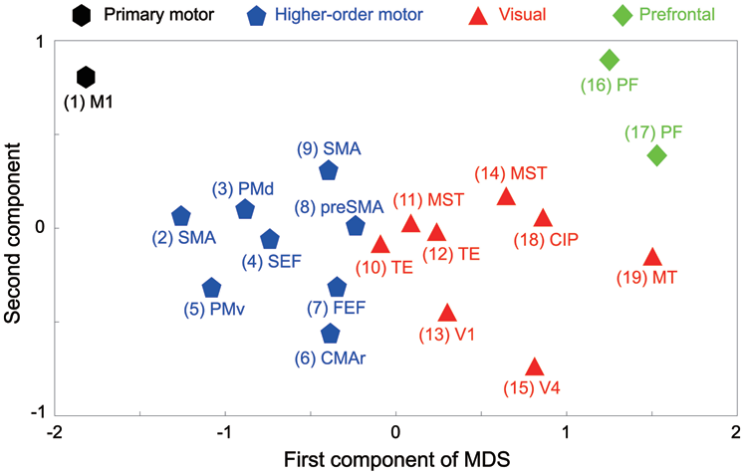
A MDS map of cortical neuronal firing based on *LvR*. The map plots the first and second components of Kruskal's nonmetric multidimensional scaling (MDS) analysis for the Hellinger distances of the metric distributions for all combinations of 19 neuronal data sets. The primary motor, higher-order motor, visual, and prefrontal areas are indicated by black hexagons, blue pentagons, red triangles, and green squares, respectively. The number in brackets next to the notation for each cortical area indicates data sets 1–19 shown in Figure 3C and Table 1.

We represented the firing characteristics of the 19 neuronal data sets as a set of (dis)similarities (Hellinger distances, cf. Materials and Methods, Equation 6) of the metric distributions across all combinations of the 19 neuronal data sets, and contracted the similarity relationship into a 2-dimensional map with Kruskal's nonmetric multidimensional scaling (MDS) analysis [42]. Figure 4 shows the 2D similarity map of the *LvR*s from the 19 neuronal data sets. The data sets are widely distributed along the first and second components, forming several clusters. The cluster (#1) for the primary motor area (M1) is at the top left, whereas those (#2–9) belonging to the higher-order motor areas (PMv, PMd, SEF, CMAr, SMA, FEF, preSMA) are near the top and to the right. The clusters (#10–15, 18–19) for the visual areas (TE, V1, MST, V4, CIP, MT) are further right and toward the bottom, and those (#16–17) for the prefrontal area (PF) are to the top and rightmost.

The first component almost exclusively represents the mean *LvR* of individual data sets. Therefore, there is a gradient of *LvR* values across the data sets corresponding to the categories of cortical functions, implying the existence of a regular-random-bursty gradient that corresponds to cortical function. The second axis is not linearly correlated to either the mean or SD of the *LvR* distribution, but the data are separated according to dissimilarities between *LvR* distributions. In particular, two PF data sets (#16, 17) are mixed with visual areas in terms of mean *LvR*, but they are isolated from the visual areas in the second axis in terms of Hellinger distance, which detects the dissimilarity of their compact *LvR* distributions from the wide *LvR* distributions of visual areas (cf. Figure 3C). Different data sets sampled from the same cortical area are arranged in the 2D MDS map relatively close to each other (cf. #16 and 17, #11 and 14, #10 and 12, and #2 and 9, see also Table 1), even though they were sampled in different laboratories using different recording methods under different experimental conditions.

### Ability of the Different Metrics to Cluster Functional Groups

In order to determine whether this functional clustering is selective to the new metric *LvR* or can also be achieved with conventional metrics, we also performed the MDS analyses for *Lv* and *Cv* (data not shown) and evaluated the goodness of the functional grouping in terms of *F*-test statistics of one-way ANOVA. *F*-values of the four functional groups (motor, higher-order-motor, visual and prefrontal areas) in MDS maps were 17.5, 9.5 and 3.0 for *LvR*, *Lv*, and *Cv*, respectively. A greater *F*-value for *Lv* than *Cv* indicates that the functional grouping is obtained using our original local variation, *Lv*, but the grouping is further improved using the revised local variation, *LvR*.

**Figure 5:**
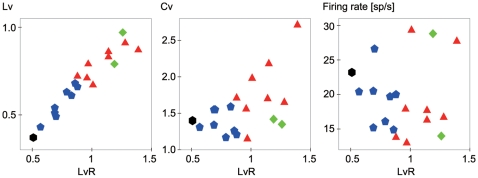
Correlation of various metrics. Mean values of *Lv*, *Cv*, and firing rate from the 19 data sets are plotted with reference to the mean *LvR*. Correlations are r = 0.95, 0.54, and 0.05, respectively. The black hexagons, blue pentagons, red triangles, and green squares represent the primary motor, higher-order motor, visual, and prefrontal areas, respectively, as described in Figure 4.

Mean values of *Lv*, *Cv*, and firing rate for the 19 data sets with reference to *LvR* values are shown in Figure 5. Their correlations are r = 0.95 (n = 19, p<0.000001), 0.54 (p<0.05), and 0.05 (uncorrelated), respectively. The degree of functional grouping in terms of these conventional metrics is observed from Figure 5.

### Relation with ISI Distributions and Firing Patterns

**Figure 6:**
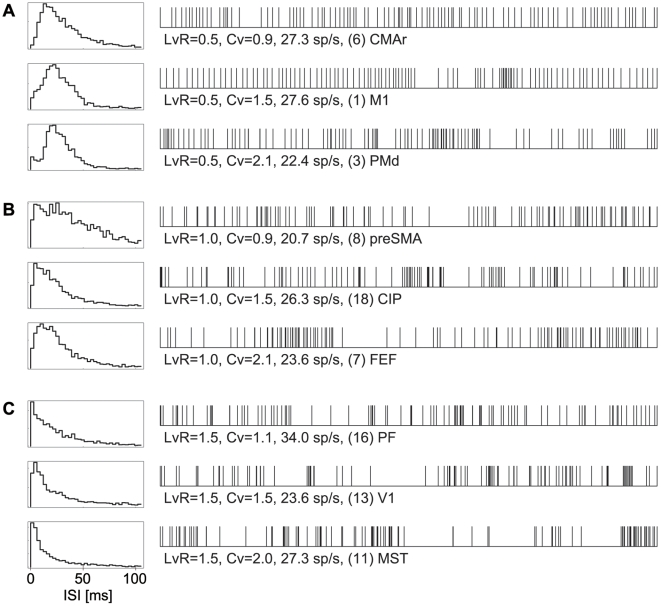
ISI distributions and sample firing patterns. (A), (B) and (C): Left: Distributions of 2,000 interspike intervals (ISIs) from neurons that exhibited *LvR* = 0.5, 1.0, and 1.5, respectively. For *LvR* values, three neurons are sampled that exhibited different *Cv* values close to 1.0, 1.5, and 2.0. Right: Sample firing patterns consisting of 100 consecutive ISIs for each.

Sample ISI distributions are shown in Figure 6 to allow for comparison with previous studies that have addressed similar questions [13], [22], [23], [53]–[55]. We sampled neurons from various cortical areas that exhibited several typical *LvR* values (close to 0.5, 1.0, and 1.5) with different *Cv* values (close to 1.0, 1.5, and 2.0), and plotted their ISI histograms and sample spike trains. The sample spike trains demonstrate that the firing patterns are captured more efficiently by *LvR* than *Cv*, and they can be characterized as regular, random, and bursty. The ISI histograms reveal that the distribution of short ISIs is correlated with *LvR*, such that short ISIs are fewer/richer for smaller/larger *LvR* sequences.

## Discussion

The major finding of this study is the existence of a regular-random-bursty gradient of intrinsic firing irregularities of cortical neurons that closely corresponds to their functional categories: primary motor, higher-order motor, visual, and prefrontal areas.

### Physiological Relevance of the Irregularity Metric

The key technique in the current analysis is a new firing metric, *LvR*, a revised form of *Lv* that strengthens detection of the intrinsic firing characteristics of individual neurons by introducing a constant, *R*, which compensates for the refractoriness effect of a previous spike. The refractoriness constant is determined by maximizing the *F*-value of the one-way ANOVA, which compares the variance of the metric means across neurons to the mean of the metric variances (Figure 2A). Refinement of the irregularity metric based on the ability to discriminate individual neurons improves functional clustering in the MDS map; the *F*-value for the four functional groups (motor, higher-order-motor, visual and prefrontal areas) is roughly doubled for *LvR* relative to *Lv*.

It is notable that the optimal *R* value of 5 ms is comparable to the known refractory period for neuronal firing [56]. Introduction of refractoriness, *R*, allows *LvR* to grasp the intrinsic firing irregularity of individual neurons with stronger invariance for firing rate fluctuation (Figure 2B and 2C). The rich variety of firing characteristics across neurons, which can be detected even after removing the firing refractory effect, implies that differences in *LvR* are not solely due to the single neuron properties, but may also be manifested by the local cortical network.

### Alternative Definitions of Firing Irregularity

Our findings indicate the presence of an innate firing regularity or irregularity with preceding spike dependency that is specific to each neuron. However, this does not seem consistent with reports that some neurons can change their firing type [57]. Three possible reasons for this apparent discrepancy are discussed below.

One possibility is that the neurons that exhibit drastic change in firing patterns are primarily interneurons. Interneurons represent a small population, thus modulation of their firing pattern, if it does occur, would not significantly affect the overall average. Modulation of firing reliability by changes in attentional conditions occurs predominantly in interneurons [58] providing support for this hypothesis.

Alternatively, changes in neuronal firing patterns may be induced experimentally by the waking to sleep transition or anaesthesia [59]. Anaesthesia was not used in our study; we measured neuronal spike sequences in awake monkeys that were performing various tasks. We did not select a particular subset of responses, rather we sampled all the available spike data, including the task periods and inter-trial intervals, between which there are significant differences in firing rates.

The third possibility is that *LvR* does not change significantly even if one class of neurons changes their firing type. Because there is not a unique definition for firing irregularity, and terms such as “bursting” and “regular”, this is a possibility. Consider for simplicity a stationary process in which ISIs are derived independently from an identical distribution. In this case, it is possible to grasp the full shape of the ISI distribution by collecting a large number of ISIs. It is, however, impracticable to characterize the full shape of the distribution function by a single or a few numerical values or a few categorical terms. For convenience, spike sequences are described by the terms “regular”, “random”, and “bursty”, as defined by the values of a metric. In principle, it is impractical for any firing pattern categorization to correspond uniquely to the conventional categories of neuronal firing, such as regular spiking, intrinsic bursting, fast spiking, or even fast-rhythmic bursting. It will be interesting to examine whether our metric of local variation, *LvR*, varies significantly with changes in firing type that are induced by current injections, anaesthesia, or sleep.

### Possible Relation of Firing Irregularities to Cell Type

In the current study, spike data were selected in a standardized manner from 19 data sets from physiological experiments with awake, behaving monkeys, solely based on the criterion that a sequence of spikes for each neuron contained greater than 2,000 spikes and the mean firing rate was greater than 5 spikes/s. Because our data do not contain information about neuronal waveforms, we could not identify the cell types of individual neurons in this study. In a previous study, we analyzed the relationship between spike waveform and firing characteristics using data from anesthetized monkeys (Figure 9 in Reference [33]). We found that neurons with thin action potentials had lower *Lv* values. Because neurons with narrow action potential waveforms are generally considered interneurons, this suggests that interneurons contribute to lowering the mean *LvR* in different areas. However, pyramidal neurons constitute the majority of neurons in cerebral cortical tissues [5] and are likely the major determinant of differences in firing characteristics in different cortical areas.

### Congruence of Spiking Patterns and Modes of Cortical Computation

In the MDS similarity map of neuronal firing irregularities, cortical areas are clustered into the categories that closely correspond to cortical functions (Figure 4). Spiking characteristics shared common traits within functional areas, even across data recorded in independent laboratories, thus indicating the presence of cortical computation–dependent mechanisms that underlie spike generation; neuronal firing is regular in the primary and higher-order motor areas, and random and bursty in the visual and prefrontal areas. Thus, the intrinsic dynamics in each cortical area may be useful for the computations specific to the functional category [5]–[12].

Firing variability measured with the Fano-factor increases as one moves from retinal ganglion cells, to the thalamic LGN and then to V1 [60]. Though this does not directly correspond to the spiking irregularities measured by *LvR*, it is tempting to assume that different signaling patterns are used depending on the level of information processing; firing variability increases as one move from sensory peripheral organs to higher-order cortical processing areas, and then decreases in the motor areas.

It seems reasonable to assume that the intrinsic regular firing in the primary and higher-order motor areas may permit real time execution of motor commands based on frequency and ensemble coding in these areas [61]. The highly irregular firing in the prefrontal and higher-order visual areas may contribute to attractor dynamics, which have been proposed to maintain working memory required for executive functions, as well as solution of ill-posed problems during various cognitive functions [2]–[4], [62]–[65].

It is also tempting to relate firing patterns to the properties of the neuronal inputs, or network parameters: It has been pointed out that a slow temporal correlation of synaptic input leads to high variability in firing [66]–[69], and irregularity of spike trains is controlled mainly by the strength of the synapses [70]. Firing in prefrontal cortical neurons is highly variable [55],[71],[72]. The present analysis with *LvR* showed that the prefrontal area is unique, in that neurons in this area rarely fire regularly, as was evidenced by the compact *LvR* distributions of two PFs in Figure 3C. This implies that there is dominance of correlated inputs in the prefrontal cortex, which may be related to the computation mode for executive functions of the prefrontal cortex.

### Merit of Analyzing Firing Patterns

Overall, our metric of the local variation of inter-event intervals provides a useful means for looking into the innate dynamics of individual neurons, as well as network dynamics, in cortical areas that may be crucial for cortical computation. We found a relation between firing patterns and cortical functions, which suggests that single-unit spike data provide information about the underlying mechanisms that may possibly include structural cues for background network connectivity. This type of cue, if further refined, may support multi-unit data analysis in revealing network structures. This method of analysis is not limited to neuronal spike sequences, rather it should be widely applicable to any sequences of signal occurrences and may help unveil and characterize mechanisms underlying signal generation.
